# Atherothrombotic Risk Factors in Patients With Rheumatoid Arthritis

**DOI:** 10.7759/cureus.59818

**Published:** 2024-05-07

**Authors:** Malu Sreekumar, Zachariah Bobby, Vir Negi, Vallayyachari Kommoju, Deepthy Sadanandan

**Affiliations:** 1 Biochemistry, Jawaharlal Institute of Postgraduate Medical Education & Research, Puducherry, IND; 2 Clinical Immunology, Jawaharlal Institute of Postgraduate Medical Education & Research, Puducherry, IND; 3 Clinical Immunology, All India Institute of Medical Sciences, Bilaspur, IND; 4 Immunology, Jawaharlal Institute of Postgraduate Medical Education & Research, Puducherry, IND; 5 Biostatistics, Jawaharlal Institute of Postgraduate Medical Education & Research, Puducherry, IND

**Keywords:** rheumatoid arthritis, biomarkers, disease activity, diagnostic predictors, atherothrombotic risk factors

## Abstract

Background: The onset of cardiovascular complications has increased the mortality rate in rheumatoid arthritis (RA) patients. Presently, there is a need to diagnose cardiovascular co-morbidity in rheumatic disease. While biomarkers such as P-selectin glycoprotein ligand-1 (PSGL-1), fibrinogen, anti-thrombin III (AT-III), hsCRP, lipoprotein (a) (lp(a)), leptin, adiponectin, and asymmetric dimethyl arginine (ADMA) are already established as independent risk factors for the development of atherosclerosis, the association of these biomarkers with disease activity in RA patients is unclear.

Methods: The case-control study comprised 40 cases along with age- and gender-matched controls recruited from a tertiary care hospital in southern India. Platelet activation in plasma was analyzed by flow cytometry using CD41 per CPCY 5.5 (platelet marker) and human CD62P FITC monoclonal antibody (P-selectin marker). Other parameters were quantified through nephelometry and ELISA. The association between the risk factors and RA disease severity, as per the disease activity score (DAS/DAS28), was analyzed. Furthermore, an ROC analysis was done to assess the utility of these biomarkers in the diagnosis of RA.

Results: With the exception of leptin, adiponectin, and ADMA, there was a significant increase in the levels of PSGL-1, fibrinogen, AT-III, hsCRP, and lp(a) when compared to healthy controls. Conventional risk factors contributing to dyslipidemia were also assessed, in which the low-density lipoprotein (LDL)/high-density lipoprotein (HDL) ratio was found to be significantly higher in RA patients compared to controls. Moreover, a significant positive correlation was identified between DAS score and activated platelets, fibrinogen, and hsCRP. ROC analysis identified that fibrinogen could predict the RA disease status with 95% accuracy, followed by activated platelets and hsCRP.

Conclusion: Several of the studied atherothrombotic risk factors were significantly altered in patients with RA. Activated platelets, fibrinogen, and hsCRP were associated with disease activity and also served as good diagnostic predictors for RA. Based on our findings, further studies could explore the potential of introducing anti-thrombotic agents in the treatment regimen of patients with RA.

## Introduction

Rheumatoid arthritis (RA) is a chronic systemic inflammatory condition that damages joints and impairs mobility [1]. Notably, in comparison with the general population, RA is emerging as a leading cause of death due to cardiovascular complications (40-50%) [2-4]. Indeed, RA has been associated with 1.3-3-fold increased mortality risk due to cardiovascular events [2-4]. Notably, study subjects with RA in a large prospective cohort of women had a significantly higher risk of myocardial infarction compared to those without RA [5]. In three-year prospectively observed patients, there was an elevated prevalence and incidence of both subclinical and clinical atherosclerosis, particularly in the subset with a disease duration of less than five years [6].

The atherosclerotic lesions in RA have been reported to develop more rapidly than usual and pose a high chance of rupture. Recent findings have uncovered new insights into the pathogenesis of atherosclerosis in RA, with the most evident one being the activation of platelets. P-selectin glycoprotein ligand-1 (PSGL-1) expressed on activated platelets has been reported to increase during inflammation and indicates inflammation intensity [7]. Despite the compelling evidence regarding the involvement of atherosclerotic pathology in the prognosis of RA (6), information on the alterations of atherothrombotic risk factors in RA patients is lacking.

Some of the prominent atherothrombotic risk factors that have been studied to prove the interplay of inflammation and thrombosis leading to increased cardiovascular morbidities are activated platelets, fibrinogen, anti-thrombin III (AT-III), hsCRP, lipoprotein (a) (Lp(a)), leptin, adiponectin, and asymmetric dimethyl arginine (ADMA). Through the modulation of inflammation, these atherothrombotic variables may influence thromboembolism risk. CRP, an acute phase reactant, was found to have direct effects on the vessel walls, and its elevation is attributed to a predisposition to atherosclerosis [8]. During the acute phase response, fibrinogen, which is a major component of a clot, is highly up-regulated by mediators like IL-6 [9]. Another marker of significance is AT-III, a crucial coagulation factor inhibitor that has been reported to influence thromboembolism risk even with minimal alterations from its basal levels [10]. ADMA, an analog of L-arginine and an inhibitor of nitric oxide, is established as an important player in endothelial dysfunction through its interaction with inflammatory cells [11]. Disruption in the balance of action in the adipocyte-derived adipokine leptin is associated with pathological circumstances like obesity and has been reported to promote vascular inflammation and vascular smooth muscle hypertrophy [12]. Adiponectin is an adipokine whose production is paradoxically decreased in obesity; however, variable results have been reported in cardiovascular diseases (CVD) [13]. Lp(a), a type of low-density lipoprotein (LDL), is another molecule known to have both atherogenic and thrombogenic properties and has been reported to activate complement pathways [14].

In RA, the presence of carotid atherosclerosis was linked to the length of the disease [15]. The intensity of RA disease activity at any given period is indicated by a disease activity score (DAS). It is determined based on a number of variables, such as lab findings, patient comments, and joint discomfort and swelling [16]. The relationship between the atherothrombotic risk factors and disease activity in RA has not been explored. The present study investigated the alteration of atherothrombotic risk factors in RA patients and assessed their association with the disease activity in RA.

## Materials and methods

Study design

The case-control study was conducted by the Department of Biochemistry, JIPMER, India in collaboration with the Department of Clinical Immunology, JIPMER, India. The study comprised 40 cases and controls who were age- and gender-matched. Cases were individuals visiting the immunology ward, who were newly diagnosed with RA as per ACR/EULAR criteria between the age group of 30 and 50. Controls were age- and gender-matched healthy volunteers not subjected to any medications related to inflammatory diseases. The study was approved by the Institute Ethics Committee (Human Studies), JIPMER (Ref no. JIP/IEC/2017/0340). Written informed consent was obtained from all adult subjects. All procedures followed were done in accordance with the Helsinki Declaration of 1975, as revised in 2013. 

Sample size calculation

The sample size was estimated using the statistical formula for comparing means for the parameter requiring a maximum number of samples. The sample size was estimated based on a previous similar study carried out in a South Indian population (8). In this study, two study groups were well-matched in terms of age and gender. The serum Lp(a) levels were 21.6+/-6.9 in the study group vs 17.2+/-4.8 in the healthy control (P=<0.001). For the present study, with an expected mean difference in Lp(a) of 4.4 mg/dL with a 95% confidence interval and 80% power, the minimum sample size estimated is 40 in each group. This was calculated using the software “PS Power and Sample Size Calculations v 3.0.”

Sample and data collection

Blood samples were collected from the study groups over a period of one year. Information regarding the patient's age, gender, height, weight, and blood pressure was obtained at a single point. The number of swollen joints and tender joints (28 joint counts) and the patient’s global assessment (one to 10 score) were estimated and a DAS28, which included CRP, was calculated. There was no follow-up with the patients for this study.

Nephelometry assay

AT-III and fibrinogen were assessed by nephelometry, through the BN Prospec system (by Siemens), in which measurement of the scattered light intensity is at a fixed angle of 13-24 degrees. N/T Protein Control PY was run for quality control check after standardizing the procedure. Plasma samples were introduced with reagents on board. About 40 μL samples were used for each test.

Assessment of parameters through autoanalyzer and ELISA

Conventional risk factor assessment was done by assessing general characteristics like BMI as well as routine parameters like blood glucose, total cholesterol, triglycerides, HDL, and LDL in clinical Beckman Coulter clinical chemistry analyzer AU 5800. hsCRP, ADMA, adiponectin, and leptin were assessed by enzyme-linked immunoassay assay (ELISA), which followed a typical two-step capture or "sandwich" type assay. The assay was carried out according to the manufacturer’s protocol. 

Flow-cytometric analysis

Platelet activation in plasma was analyzed by flow cytometry (BD FACSCalibur) using CD41 per CPCY 5.5 (platelet marker) and Human CD62P FITC monoclonal antibody (P-selectin marker). P-selectin can be detected using flow cytometry using a P-selectin antibody, which is fluorochrome tagged. Using the appropriate gating methodology, the percentage activation of platelets can be determined from these gated cells. Fluorescence signal strength is determined and equated to the platelet activation percentage.

Estimation of platelet activation

Platelet activation in plasma was analyzed by flow cytometry (BD FACSCalibur) using CD41 per CP Cy 5.5 (platelet marker) and Human CD62P FITC monoclonal antibody (P-selectin marker). Platelet-rich plasma was obtained by centrifuging at 300 rpm for 30 minutes. Subsequently, 100 µL of the sample was aliquoted onto a corning falcon tube. To the tubes containing cells, 10 µL of CD41 per CP and CD 62P FITC were added and incubated in the dark for one hour. The sample was then diluted with 750 µL of PBS and analyzed directly in BD FACSCalibur using two lasers 488 nm and 635 nm. Three color calibrate beads were used for calibration prior to sample processing for which the software FACS Comp was used. Cell Quest Pro software was used for the acquisition and analysis of samples. FACS clean and distilled water were used for cleaning after sample processing.

Statistical analysis

Normally distributed variables were represented as mean with standard deviation. Variables of non-normal distribution were represented as median with interquartile range. Independent students t-test and Mann-Whitney U test were used to compare the differences between group means. Pearson’s correlation analysis was used to assess the association between the atherothrombotic risk factors and the DAS score. Spearman’s rank correlation was used to assess the relation of non-gaussian data with the DAS score. The association between two categorical variables was analyzed by the Chi-square test. All statistical analyses were carried out for two-tailed significance and a p-value of ≤0.05 was considered as statistically significant. All statistical analysis was carried out using SPSS version 20.

## Results

Over a period of one year, 40 cases and controls were recruited based on the inclusion and exclusion criteria. Females constituted 85% (N=34) of the cases and 67% (N=27) of the controls. Information regarding the patient's age, gender, height, weight, and blood pressure was obtained at a single point. The mean age of cases and controls were 47.7 and 48.3, respectively (Table 1). The number of swollen joints and tender joints (28 joint counts) and the patient’s global assessment (one to 10 score) were estimated and a DAS28, which included CRP, was calculated. All participants completed a health assessment questionnaire, which was essentially a survey including any previous CVD event as defined by acute myocardial infarction, stroke, or coronary artery bypass surgery, and a survey of CVD risk factors and lifestyle.

**Table 1:** General characteristics and lipid profile among patients with RA in comparison to healthy control ^$^Mann Whitney U test was done, ^#^A Chi-square test was done, and for all other variables, an independent t-test was done *P-value <0.05 - statistical significance Age, LDL-C, HDL-C, total cholesterol, LDL/HDL, FBS, and BMI are represented as mean±SD. Gender is represented as frequency (%); TG and VLDL are represented as median (Q1, Q3). RA, rheumatoid arthritis; TG, triglyceride; LDL, low-density lipoprotein; HDL, high-density lipoprotein; FBS, fasting blood sugar; BMI, body mass index; VLDL, very low-density lipoprotein; LDL-C, low-density lipoprotein-cholesterol; HDL-C, high-density lipoprotein-cholesterol

Parameters	Controls (Mean±SD/Median (Q1,Q3)/Frequency (%))	Cases (Mean±SD/Median (Q1,Q3)/Frequency (%))	P-value
Age	48.30±5.61	47.75±5.64	0.66
Males^#^	13 (32.50)	6(15)	0.07
Females^#^	27 (67.5)	34(85)	
TG^$^ (mg/dL)	84 (65.75, 134.25)	98( 86, 142.75)	0.05
LDL-C (mg/dL)	83.85±20.23	91.65±19.68	0.08
HDL-C (mg/dL)	30.72±7.18	30.18±6.32	0.71
Total Cholesterol (mg/dL)	131.82±33.03	144.922±28.13	0.06
VLDL-C^$^ (mg/dL)	17 (13.25,26.75)	20 (17.25,28.75)	0.05
LDL-C/HDL-C (mg/dL)	2.7820±0.6080	3.1242±0.7921	0.03*
FBS (mg/dL)	90.18±24.64	104.65±39.26	0.05
BMI (kg/m^2^)	22.58±2.58	22.73±2.44	0.79

Comparison of traditional risk factors like BMI and lipid profile

There was no significant difference in the BMI between the two groups. There was an impairment in the fasting glucose levels among the patients of RA when compared to the healthy controls. The low-density lipoprotein-cholesterol/high-density lipoprotein-cholesterol (LDL-C/HDL-C) ratio was found to be higher among the cases when compared to controls. There was a trend toward an increase in parameters of lipid profile among patients of RA. However, it did not reach statistical significance. These results are presented in Table 1.

Comparison of atherothrombotic risk factors between patients of RA and healthy controls

There was a significant increase in the levels of all the estimated atherothrombotic risk factors when compared to healthy controls except Lp(a), leptin, and ADMA. However, there was a trend toward an increase in ADMA levels among RA patients. The results are presented in Table 2.

**Table 2:** Comparison of atherothrombotic risk factors between patients with RA and healthy controls *P-value <0.05 - statistical significance The mean and SD of all parameters are provided in the table except ADMA, which is presented as median (Q1,Q3) RA, rheumatoid arthritis; AT-III, anti-thrombin III; ADMA, asymmetric dimethyl arginine; Lp(a), lipoprotein (a)

Parameters	Controls (Mean±SD/Median (Q1,Q3))	Cases (Mean±SD/Median (Q1,Q3))	P-value
Activated Platelets (%)	57.95±20.08	77.17±11.33	<0.001^*^
Fibrinogen (gm/L)	2.34±0.69	4.16±1.11	<0.001^*^
AT-III (gm/L)	0.2411±0.0533	0.2907±0.0472	<0.001^*^
hsCRP (ng/mL)	3140.52 (1517.69,7330.90)	9294.41 (8520.26,11423.02)	0.027^*^
Lp(a) (ng/mL)	8.82 (4.56,14.83)	7.67 (4.64,12.46)	0.146
Leptin (ng/mL)	11.06 (6.02,28.12)	8.62 (4.56,16.66)	0.102
Adiponectin (ng/mL)	22.83±4.16	20.84±4.33	0.039^*^
ADMA (ng/mL)	88.72 (69.90,158.40)	96.93 (71.18,155.73)	0.617

FACS analysis of P-selectin on activated platelets

**Figure 1:**
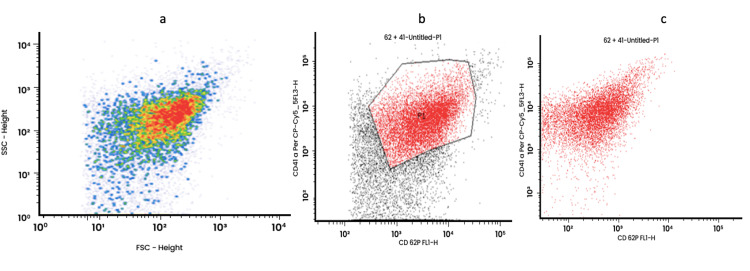
a: Dot plots by SSC (Y-axis) and FSC (X-axis). b: Dot plots by SSC (Y-axis) and FSC x (X-axis). c: Cells stained positive for CD41 and CD62 FSC, forward scatter; SSC, side scatter

Platelet activation in plasma was analyzed by using CD41 per CPCy 5.5 (platelet marker) and Human CD62P FITC monoclonal antibody (P-selectin marker). CD62P (P-selectin expressing platelet population) was isolated from a population of CD41 expressing population by gating. Appropriate gating was established by using an FMO sample. There was a significant difference between the CD41+CD62P+ population in RA patients and controls. CD41+CD62P+ was assessed as percentage activation. This is illustrated in Figure 1a-1c.

Diagnostic utility of atherothrombotic risk factors in RA

**Figure 2:**
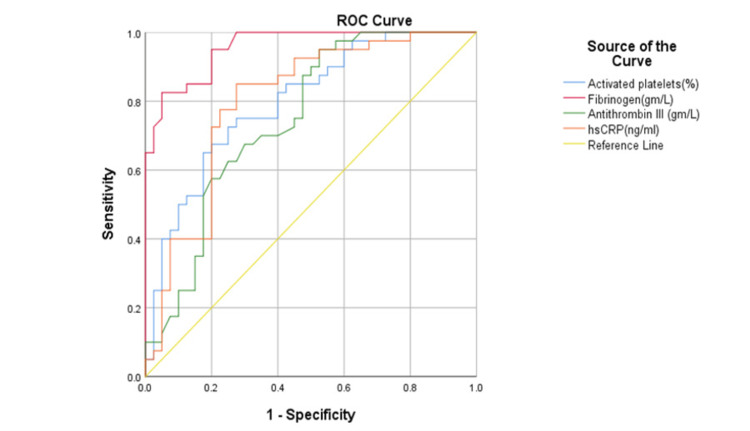
ROC curve analysis for the differential diagnosis of RA based on atherothrombotic risk factors RA, rheumatoid arthritis

The utility of atherothrombotic risk factors in the diagnosis of RA was assessed by ROC analysis. The area under the curve (AUC) was found to be highest for fibrinogen (0.957) followed by hsCRP and activated platelets. The details are provided in Table 3 and Figure 2. Patients were diagnosed with RA by clinical findings, which included no swollen joints (DAS28) and CRP.

**Table 3:** Utility of atherothrombotic risk factors in the diagnosis of RA by ROC analysis RA, rheumatoid arthritis; AT-III, anti-thrombin III; AUC, area under the curve

Parameters	Cut Off	Sensitivity	Specificity	AUC (95% CI)
Activated Platelets (%)	69.55	75%	73%	0.798 (0.701,0.894)
Fibrinogen (gm/L)	2.885	95%	80%	0.957 (0.920,0.994)
AT-III (gm/L)	0.2675	70%	62.5%	0.748 (0.640,0.856)
hsCRP (ng/mL)	8882.43	72.5%	80%	0.800 (0.700,0.900)
Leptin (ng/mL)	8.84	50%	40%	0.394 (0.270,0.518)
Adiponectin (ng/mL)	21.43	50%	35%	0.374 (0.251,0.498)

Correlation analyses between atherothrombotic risk factors and disease severity by DAS score

**Figure 3:**
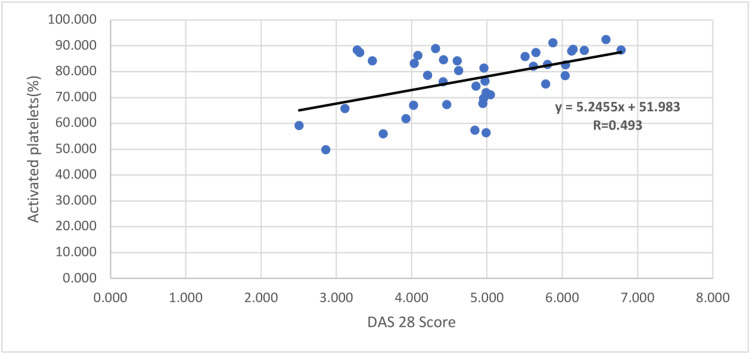
Scatter plot analysis between DAS28 score vs activated platelets among rheumatoid patients DAS, disease activity score

**Figure 4:**
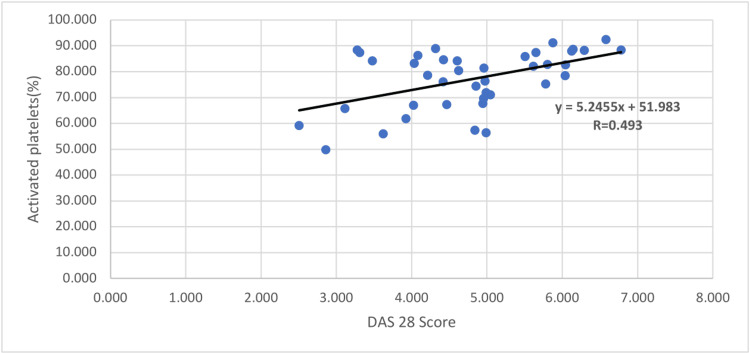
Scatter plot analysis between DAS28 score vs fibrinogen among rheumatoid patients DAS, disease activity score

**Figure 5:**
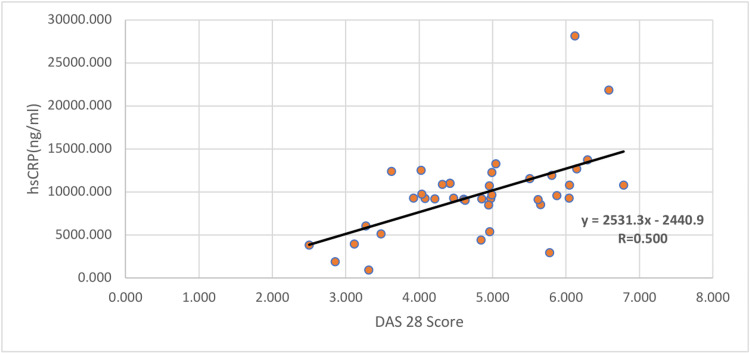
Scatter plot analysis between DAS28 score vs hsCRP among rheumatoid patients DAS, disease activity score

Correlation analyses between DAS score and atherothrombotic risk factors showed a statistically significant positive correlation with a few parameters like activated platelets, fibrinogen, and hsCRP. HsCRP had a correlation coefficient of 0.50 (p-value of <0.001). Activated platelets had a correlation coefficient of 0.493 (p-value of <0.001) and fibrinogen had a correlation coefficient of 0.414 and p-value of 0.008. This is explained in Table 4 and Figures 3-5.

**Table 4:** Correlation analyses between atherothrombotic risk factors and DAS score among patients with RA ^$^Pearson correlation ^#^Spearman's rank correlation *P-value <0.05 - statistical significance AT, thrombin; ADMA, asymmetric dimethyl arginine; Lp(a), lipoprotein (a); DAS, disease activity score

Parameter 1	Parameter 2	Pearson Correlation Coefficient (r)	P-value
DAS score	Activated platelets^$^	0.493	<0.001*
	Fibrinogen^$^	0.414	0.008*
	hsCRP^#^	0.500	<0.001*
	AT^$^	-0.202	0.212
	Lp(a)^#^	-0.097	0.550
	Leptin^#^	-0.045	0.784
	Adiponectin^$^	-0.155	0.340
	ADMA^#^	-0.012	0.940

## Discussion

RA is associated with an increase in mortality of 1.3-3-fold and is emerging as a leading cause of death owing to cardiovascular complications. Atherosclerosis in RA is primarily linked to inflammation, which plays an important role in its initiation, perpetuation, and exacerbation, through the modulation of traditional risk factors and direct effect on blood vessel walls [17]. Apart from the traditional risk factors, our current research aimed to explore the association of atherothrombotic risk factors like P-selectin, fibrinogen, AT-III, lp(a), hsCRP, leptin, adiponectin, and ADMA with disease severity in RA patients. Disease severity was assessed by DAS28 and checked for positive correlations with the newer biomarkers. We observed that there was a significant increase in the levels of PSGL-1, fibrinogen, AT-III, hsCRP, and lp(a) in RA patients when compared to healthy controls. LDL-C/HDL-C ratio was also found to be significantly higher in RA patients compared to controls. Moreover, we found a marked positive association between DAS score and activated platelets, fibrinogen, and hsCRP. Furthermore, our ROC analysis identified that fibrinogen could predict the RA disease status with 95% accuracy, followed by activated platelets and hsCRP. 

Platelet activation markers and platelet-derived microparticles have been reported to behave not only as triggers of arthritis but also form an essential link between RA and accelerated atherosclerosis [7]. Indeed, activated platelets express certain adhesion receptors, which subsequently contribute to atherosclerotic plaque formation [18]. P-selectin, an activated platelet marker (CD62P), is rapidly translocated from platelet granules as well as from Weibel-Palade bodies of endothelial cells toward the cell surface on platelet activation. During the process of platelet aggregation, the glycoprotein GP IIb/IIIa on platelets undergoes an activation-dependent conformational change, which effectively helps to bind soluble fibrinogen [19]. Subsequently fibrinogen cross-links platelets by bridging GP IIb/IIIa in between adjacent platelets, eventually forming platelet aggregates. The progressive stabilization of these platelet-fibrinogen interactions leads to thrombotic events, which play a pivotal role in atheroma formation and plaque rupture. 

In the present study, the percentage of activated platelets in RA patients was assessed from CD62P and CD41 positive population by FACS analysis and compared with healthy controls. We noted that activated platelets were significantly higher in RA patients compared to control subjects (p-value <0.001) in line with the evidence from our study by Vona et al., which identified markedly elevated platelet aggregation in RA women compared to healthy individuals [20]. Moreover, treatment with disease-modifying anti‐rheumatic drugs (DMARDs) significantly reduced platelet aggregation, indicating the marked involvement of platelet activation with RA pathogenesis [20]. Likewise, in the present study, we identified that activated platelets correlated positively with disease activity in RA. Furthermore, to study the utility of these markers, we performed a ROC analysis and noted that the sensitivity and specificity of the activated platelets were 75% and 73%, respectively, with a cut-off of 69.55% with the AUC value of 80%, indicating the good discriminative ability of activated platelets in predicting RA disease. Elevated concentrations of von Will brand factor, fibrin D-dimer, and tissue plasminogen activator antigen also have some salient associations in RA patients [21]. A follow-up study including these parameters is warranted to further our understanding of atheroma formation in RA patients.

Increased levels of fibrinogen have been noted in the synovial fluid of RA patients [22]. A meta-analysis of fibrinogen levels and subsequent vascular pathologies reported that a 100 mg/dL increase in fibrinogen levels with a hazard ratio of 1.8 can lead to coronary heart disease after adjusting for other conventional risk factors and CRP [23]. One of the aims of our study was to compare circulating fibrinogen levels in RA patients with levels in healthy controls and to explore relationships with clinical disease activity and acute-phase markers. In our study, fibrinogen was significantly elevated in RA patients when compared to healthy controls. Some of the previous studies on RA suggested that plasma fibrinogen levels seem to correlate well with DAS28 [24]. In the present study, we noted that elevated fibrogenesis was associated with higher disease activity in RA patients. The diagnostic utility of fibrinogen in RA patients was also explored using ROC, and we identified that the fibrinogen is able to predict the disease status 95.7% accurately. It is to be noted that the sensitivity and specificity of the fibrinogen are 95% and 80% at a cut-off of 2.885 g/L. As the role of fibrinogen is increasing in coronary vascular events as established from previous studies, it is emerging as a predictive novel marker of CVD in RA patients. 

AT-III is one of the most crucial coagulation factor inhibitors that is seen to suppress inflammation through both coagulation-dependent and independent effects [25]. AT-III executes its anti-inflammatory action by two major mechanisms, which include the inhibition of factor Xlla and kallikrein, which activate neutrophils and prevent organ damage, and the inhibition of thrombin, which plays a major role in the inflammatory response by endothelial cell activation [25]. It has been found that even mild AT-III deficiency was able to predict risk for thromboembolism [26]. In the present study, we noted a highly significant increase in AT-III levels in RA patients. This could be attributed to the compensatory increase to curb the inflammatory and prothrombotic microenvironment. Few studies have reported an increase during the early stages of inflammation [27]. Moreover, Jones et al. noted that even though AT-III is elevated in synovial fluid of RA patients, there was a depression in its activity compared with matched controls, indicating its inactivation in RA joints [28]. Further studies that investigate the relevance of AT-III at different time points of RA disease and its expression in response to treatment are warranted.

CRP has direct inflammatory effects on the blood vessel wall, which predisposes its risk to atherosclerosis [29]. Elevated CRP predominantly mediates the atherosclerotic effect by stimulating the release of cellular adhesion molecules by vascular endothelial cells. This facilitates the adhesion and migration of monocytes through the vessel wall. Subsequently, it increases the uptake of LDL cholesterol by macrophages, leading to the activation of complement pathways [30]. In our study, hsCRP was found to be significantly elevated (p-value=<0.001) in RA patients and showed a positive correlation with RA disease activity (r=0.500; p-value=<0.001). On assessing its diagnostic utility as a marker, the AUC was found to be 0.800 with specificity and sensitivity of 72.5% and 80%, respectively. Miguel et al. reported that elevated CRP in RA patients was associated with major indicators of generalized atherosclerosis [31]. Larger prospective follow-up studies could further underline the potential of CRP as a potent marker for atherosclerotic risk in RA patients. 

The apolipoprotein moiety (apo(a)) increases the atherogenic properties of Lp(a), is based on apo(a) size mostly, and is genetically predetermined. Heterogeneity in apo(a) size is related to the variable number of the protein domains, the Kringle IV type 2, which has an inverse relationship with density and plasma concentration of Lp(a) [32]. Individuals with smaller apo(a) are found to have the highest Lp(a) concentrations, thereby greater cardiovascular risk. Though plasma concentrations of Lp(a) exhibit a high genetic predisposition, higher concentrations of Lp(a) have been observed in the backdrop of inflammation also. A previous study reported higher concentrations of Lp(a) in RA patients, even though they did not observe a marked change in the levels of cholesterol, LDL, and VLDL between RA and control subjects [33]. In our study on a south Indian population, we did not find a significant difference in Lp(a) levels between RA and control subjects, even though there was a trend toward an increase in RA patients. Moreover, in the present study Lp(a) levels did not correlate with RA disease activity. Furthermore, studies are warranted to understand whether the estimation of Lp(a) can be a useful tool in the risk assessment of RA patients for atherosclerosis risk, in addition to traditional lipid profile estimation.

There is ample evidence that proves that leptin acts as a proinflammatory cytokine in immune responses such as RA [34]. Dysregulation of leptin exerts harmful effects by precipitating joint inflammation synergistically with IL-1 and IFN-γ [34]. RA patients having erosive joint disease are found to have higher leptin concentrations than those without erosions, suggesting that leptin plays a key role in gradual joint destruction [34]. Of interest, in the present study, leptin levels were found to be reduced in RA patients compared to control subjects. Furthermore, the leptin levels did not correlate with disease activity in RA patients. Popa et al. reported that leptin concentration was inversely associated with inflammation in RA patients [35]. This shows that plasma leptin levels may decrease if chronic inflammation as prevalent in RA is ongoing. Whether lower leptin in RA patients is an adaptive mechanism needs further investigation.

Production as well as effects of adipokines such as adiponectin can be altered in patients with autoimmune diseases like RA [36]. Studies have indicated that adiponectin exhibits antidiabetic and vasculo-protective effects [37]. Inverse or favorable and direct or paradoxically unfavorable associations of adiponectin concentrations were observed in many studies attributing to the metabolic risk factors in RA [38-40]. In our study adiponectin was found to be decreased in RA patients compared to controls. Furthermore, no correlation was found between adiponectin levels and disease severity. One study reported that serum adiponectin levels improved after treatment with DMARDs in RA patients, suggesting that active inflammation as seen in RA may decrease circulating adiponectin levels [41]. A different study suggested that the leptin:adiponectin ratio could be used to assess the common carotid artery resistive index and thus predict cardiovascular outcomes in RA patients [42]. Future studies could consider using the leptin:adiponectin ratio in the assessment of cardiovascular risk in RA patients.

In the recent past, many studies have investigated the role of ADMA and its usefulness as a predictive marker for estimating cardiovascular risks. Recently, a meta-analysis comprising 20,000 non-overlapping participants enrolled in 22 cohort studies and their long-term follow-up proved an association between ADMA levels and cardiovascular events [43]. Various studies showing an association between markers of disease activity and ADMA in RA led to conflicting results [44]. In the present study, we observed no significant difference in ADMA levels between RA patients and controls. Furthermore, we did not observe any correlation between ADMA and RA disease activity. While Krochin et al. demonstrated in RA patients the existence of a positive correlation between ADMA levels, fibrinogen, CRP, and disease activity, suggesting a link between ADMA levels, high inflammatory state, and CVD in RA, other studies failed to support these observations [45-47]. In line with our findings, Erre et al. did not identify any difference in ADMA levels in RA patients and further noted the absence of an association between arterial stiffness and ADMA in these patients [46]. Further studies with lesser heterogeneity and a larger population might reveal if ADMA could serve as an atherosclerosis risk marker in RA patients. 

## Conclusions

We found a significant increase in many of the atherothrombotic risk factors among the RA patients of our study. Not many reports are available from the Indian population on the levels of atherothrombotic risk factors. Hence our study assumes significance in regard to the fact that Indians are more prone to develop premature coronary heart diseases. We also observed that 85% of our cases comprised women with a mean age of 47 years. This suggests that RA is associated with an increased risk of coronary artery disease at a younger age among women who are considered to be cardioprotective. In view of the observations of the present study, further studies could explore the potential of introducing anti-inflammatory agents and anti-thrombotic agents in the treatment regimen of patients with RA.
